# Excite Spoof Surface Plasmons with Tailored Wavefronts Using High‐Efficiency Terahertz Metasurfaces

**DOI:** 10.1002/advs.202000982

**Published:** 2020-08-05

**Authors:** Zhuo Wang, Shiqing Li, Xueqian Zhang, Xi Feng, Qingwei Wang, Jiaguang Han, Qiong He, Weili Zhang, Shulin Sun, Lei Zhou

**Affiliations:** ^1^ State Key Laboratory of Surface Physics Key Laboratory of Micro and Nano Photonic Structures (Ministry of Education) Fudan University Shanghai 200433 China; ^2^ Shanghai Engineering Research Center of Ultra‐Precision Optical Manufacturing Green Photonics and Department of Optical Science and Engineering Fudan University Shanghai 200433 China; ^3^ Center for Terahertz Waves College of Precision Instrument and Optoelectronics Engineering Tianjin University Key Laboratory of Optoelectronics Information and Technology (Ministry of Education) Tianjin 300072 China; ^4^ Academy for Engineering and Technology Fudan University Shanghai 200433 China; ^5^ Collaborative Innovation Center of Advanced Microstructures Nanjing 210093 China; ^6^ School of Electrical and Computer Engineering Oklahoma State University Stillwater OK 74078 USA

**Keywords:** metasurfaces, near field, plasmonic couplers, spoof surface plasmons, wavefronts

## Abstract

Spoof surface plasmons (SSPs) play crucial roles in terahertz (THz) near‐field photonics. However, both high‐efficiency excitation and wavefront engineering of SSPs remain great challenges, which hinder their wide applications in practice. Here, a scheme is proposed to simultaneously achieve these two goals efficiently using a single ultracompact device. First, it is shown that a gradient meta‐coupler constructed by high‐efficiency Pancharatnam–Berry (PB) meta‐atoms can convert circularly polarized (CP) THz beams into SSPs with absolute efficiency up to 60%. Encoding a parabolic phase profile into the meta‐coupler based on the PB mechanism, it is demonstrated that the device can covert CP beams into SSPs with focusing or defocusing wavefronts, dictated by the chirality of the incident wave. Finally, two distinct chirality‐dependent phase distributions are encoded into the meta‐coupler design by combining the PB and resonance phase mechanisms, and it is demonstrated that the resulting meta‐device can achieve SSP excitations with chirality‐delinked bifunctional wavefront engineering. THz near‐field experiments are performed to characterize all three devices, in excellent agreement with full‐wave simulations. The results pave the road to realize ultracompact devices integrating different functionalities on near‐field manipulations, which can find many applications (e.g., optical sensing, imaging, on‐chip photonics, etc.) in different frequency domains.

## Graphical abstract

High‐efficiency bifunctional manipulations on surface‐plasmon wavefronts are enabled by a single ultracompact meta‐device. Constructed with meta‐atoms possessing both geometric and resonant phases, the meta‐device exhibits distinct phase profiles for impinging lights with different spins, thus generating independent surface‐plasmon profiles. These findings may find many applications in integration photonics, such as coupling on‐chip optical devices and enhancing light–matter interactions.

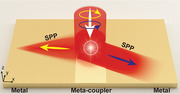

## 1 Introduction

Surface plasmons (SPs) are eigen electromagnetic (EM) modes bounded at dielectric/metal interfaces coupled with free‐charge oscillations inside the metal.^[^ ^1^ ^]^ Two extraordinary properties of SPs (i.e., subwavelength lateral resolution and strong field enhancement at interfaces) offer them many attractive applications, such as super‐resolution imaging,^[^ ^2^ ^]^ subwavelength nanocircuits,^[^ ^3^ ^]^ bio‐ or chemical sensing,^[^ ^4^, ^5^ ^]^ enhanced nonlinear^[^ ^6^, ^7^ ^]^ or Raman effects,^[^ ^8^, ^9^ ^]^ plasmonic laser,^[^ ^10^ ^]^ and so on. However, SPs do not naturally exist in low‐frequency regimes (e.g., microwave and terahertz (THz)) where metals are too conductive. Decades ago, guided surface waves or other types of eigen EM waves were already found to exist on structured metallic surfaces even in the microwave regime.^[^ ^11^, ^12^, ^13^, ^14^, ^15^, ^16^, ^17^, ^18^, ^19^, ^20^, ^21^ ^]^ These eigen EM modes on structured metals are now generally termed as spoof surface plasmons (SSPs),^[^ ^22^ ^]^ which exhibit similar characteristics as natural SPs but with properties dictated by the artificial structures rather than constitutional materials. Recently, the concept of SSP was further extended to the THz regime, which has found rich applications in THz near‐field photonics.^[^ ^23^, ^24^, ^25^, ^26^, ^27^ ^]^

**Figure 1:**
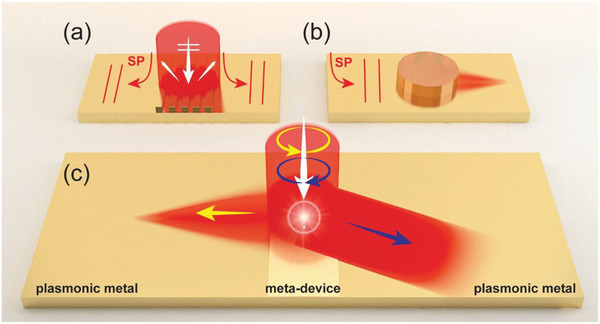
Schematics of the high‐efficiency chirality‐delinked surface‐plasmon (SP) meta‐device. a) Schematics of SP excitations by a grating coupler. b) Schematics of SP focusing by a cylindrical dielectric disk. c) Schematics of a single meta‐device achieving SP excitations and incident‐chirality‐delinked wavefront tailoring with high efficiencies.

In realizing these fascinating applications, it is necessary to efficiently excite the SPs (including SSPs at low frequencies) and modulate the wavefronts of the generated SPs as desired. Unfortunately, these two operations are usually performed on different devices with low efficiencies. For example, typically one employs a prism^[^ ^28^, ^29^, ^30^ ^]^ or grating coupler^[^ ^31^, ^32^ ^]^ to first excite an SP beam (**Figure** **1**a) on a metallic surface, and then uses other on‐chip devices, constructed based on similar concepts with their free‐space counterparts, to modulate the wavefronts of the generated SP beams (see Figure 1b) based on the application requests (e.g., focusing, reflection, and refraction).^[^ ^33^, ^34^ ^]^ However, both devices constructed with natural materials are of large lateral sizes and low working efficiencies, not mentioning that combining them together could occupy a larger on‐chip space and further lower the final efficiency.

Recently, metasurfaces (ultrathin metamaterials composed by planar subwavelength microstructures with tailored optical responses) were shown to exhibit powerful capabilities to manipulate EM waves.^[^ ^35^, ^36^, ^37^, ^38^, ^39^ ^]^ Through encoding purposely designed phase profiles to metasurfaces for transmitted/reflected waves, many fascinating wave‐manipulation effects were demonstrated based on Hyugens’ principle, such as anomalous light refraction and reflection,^[^ ^35^, ^36^, ^40^ ^]^ high‐efficiency excitations of SPs and SSPs,^[^ ^37^, ^41^, ^42^ ^]^ meta‐lens imaging^[^ ^43^, ^44^ ^]^ and holograms,^[^ ^45^, ^46^ ^]^ and information coding.^[^ ^47^ ^]^ Phase responses of meta‐atoms can be tailored by the resonance mechanism^[^ ^35^, ^36^, ^37^ ^]^ and/or the Pancharatnam–Berry (PB) one,^[^ ^48^, ^49^ ^]^ typically corresponding to linearly polarized (LP) and circularly polarized (CP) light excitations, respectively. Therefore, metasurfaces constructed with PB meta‐atoms can manipulate CP waves efficiently, yielding fascinating effects such as the photonic spin‐Hall effect,^[^ ^50^, ^51^, ^52^ ^]^ spin controlled SP excitations,^[^ ^49^, ^53^ ^]^ polarization‐insensitive cloaking,^[^ ^54^ ^]^ and so on. However, despite great successes already achieved with metasurfaces, so far the two SP‐control functionalities, i.e., excitation and wavefront engineering, are often realized with separate meta‐devices, which are unfavorable for integration‐optics applications. While a holographic technique was utilized to design PB meta‐devices for generating two spin‐controlled SP profiles,^[^ ^55^ ^]^ such devices suffer from the low‐efficiency issue and the SP‐control bifunctionalities are only meaningful inside a specific region.

In this article, we demonstrate a scheme to efficiently launch SSPs with well‐controlled wavefronts using a single meta‐device, based on three successive experiments in the THz regime. We first show that a gradient‐phase meta‐coupler, constructed with purposely designed high‐efficiency PB meta‐atoms, can achieve chirality‐modulated directional SSP excitations with absolute efficiency up to 60% at 0.4 THz. We next use the same PB meta‐atom to realize another meta‐coupler encoded with gradient and parabolic phase profiles along two directions, and then demonstrate that it can generate SSP beams with chirality‐locked wavefront modulations. Finally, we combine PB and resonance mechanisms to realize a meta‐coupler that can convert incident CP waves to SSP beams with chirality‐delinked wavefront modulations. THz near‐field scanning experiments are in excellent agreement with full‐wave simulations on all three cases studied, which collectively demonstrate the validity of our scheme.

## 2 Results and Discussions

### 2.1 Design and Characterization of a High‐Efficiency PB Meta‐Atom

We start from designing a high‐efficiency PB meta‐atom in the THz regime. Consider a reflective meta‐atom exhibiting mirror symmetry with optical properties described by a Jones matrix $R=(\begin{array}{cc} r_{uu} & 0\\ 0 & r_{vv} \end{array})$, where *u* and *v* denote the two principle axes, respectively. As discussed by Luo et al.,^[^ ^50^, ^51^ ^]^ any meta‐device constructed by such PB meta‐atoms always generate two reflective beams upon illumination of a CP wave with a particular chirality, i.e., a normal beam and an anomalous one, exhibiting opposite and identical chirality with the incident one, respectively. The normal beam is just a specularly reflected one with efficiency *R_n_* = |*r_uu_* + *r_vv_*|^2^/4, while the anomalous beam is the desired one that is reconstructed from the interference among waves scattered by different meta‐atoms. Therefore, the working efficiency of our device is solely dictated by the intensity of the anomalous beam, *R_a_* = |*r_uu_* − *r_vv_*|^2^/4. Neglecting material losses, we find that the criterion to achieve a 100% efficiency meta‐device is

(1) $$\begin{array}{c} \left|r_{uu}\right|=\left|r_{vv}\right|=1,\;\arg\left(r_{uu}\right)-\arg\left(r_{vv}\right)=\pi \end{array}$$

indicating that our meta‐atom should behave as an ideal half‐wave plate.^[^ ^50^ ^]^ With material losses taken into account, the efficiency cannot reach the theoretical limit of 100%, but Equation (1) is still meaningful to guide us search for the best PB meta‐atom with maximum efficiency.

**Figure 2:**
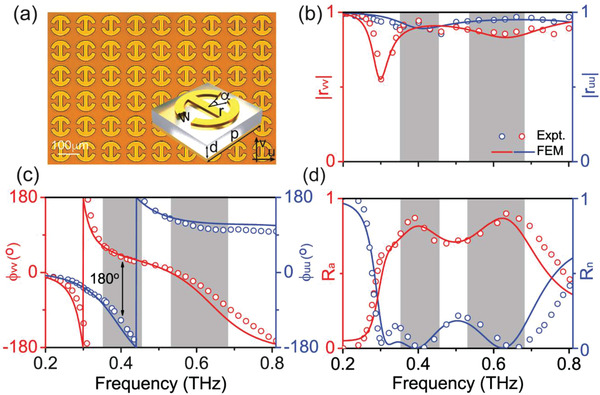
Design and characterization of a high‐efficiency PB meta‐atom. a) Picture of part of a fabricated metasurface consisting of a periodic array of PB meta‐atoms with geometry shown in the inset. Here, *r* = 43.3 µm, *w* = 23.2 µm, *α* = 33°, and *p* = 166.6 µm. Measured and stimulated spectra of reflection b) amplitude and c) phase of the fabricated sample for $\vec{E}\left|\right|\hat{u}$ and $\vec{E}\left|\right|\hat{v}$ polarizations, respectively. d) Measured and simulated efficiencies of the anomalous and normal modes (R_a_ and *R*_n_), determined by the Jones matrix characteristics of the meta‐atoms.

Inset to **Figure** **2**a depicts the geometry of our PB meta‐atom designed with the help of Equation (1), which is in metal‐insulator‐metal configuration composed of a gold connected‐double‐ring resonator and a gold mirror separated by a 60 µm‐thick quartz spacer (ɛ_r_ = 3.9 + 0.11*i*). We fabricate a sample consisting of a periodic array of such meta‐atoms, and then use THz time domain spectroscopy (TDS) to characterize the optical properties of sample. The gold film on the bottom blocks any transmissions through the sample, and thus we only need to measure the reflection spectra of the sample. Open circles in Figure 2b,c depict the measured spectra of reflection amplitude and phase of the sample, under the illuminations of THz lights polarized along *u* and *v* axes, respectively. The dips in two spectra (at 0.3 and 0.43 THz) correspond to two magnetic resonances supported by the system for two polarizations, associated with strongly varying reflection phases covering nearly a 2*π* range (see Figure 2c). The position of these two resonances is carefully chosen (via optimizing the geometric structure of the meta‐atom) so that the phase difference Φ*_uu_* – Φ*_vv_* is kept at about *π* within a broad frequency band (0.3–0.65 THz). Furthermore, we also optimized the spacer thickness to let two magnetic resonances locate in the under‐damped regime,^[^ ^56^ ^]^ which results in weak absorptions in two polarizations (see Figure 2b). Put the measured reflection amplitudes and phases to Equation (1), we computed the efficiencies of two modes (*R*_n_ and *R*_a_) of our PB meta‐atom, and depicted the results in Figure 2d. Clearly, the absolute working efficiency of our meta‐atom is significantly enhanced within two frequency bands centered at 0.4 and 0.63 THz, with peak values reaching 0.86 and 0.9, respectively. If we define a relative working efficiency as *R*_a_/(*R*_n_ + *R*_a_), we find that the maximum value of relative efficiency can approach ≈100%, indicating that losses are the key factor to suppress the absolute working efficiency. Full‐wave simulations using a finite‐element‐method (FEM) are in excellent agreement with all experimental results.

### 2.2 High‐Efficiency Directional SSP Meta‐Coupler

**Figure 3:**
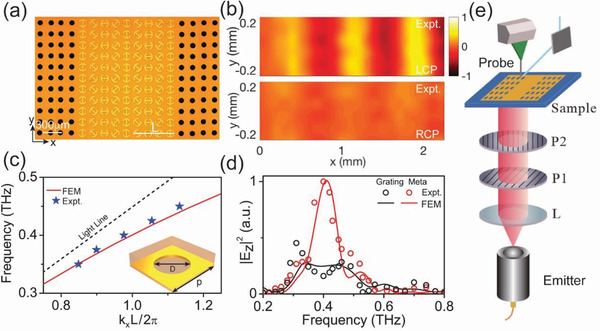
Near‐field characterizations on the THz PB meta‐coupler. a) Picture of part of the fabricated meta‐coupler composed by a PB gradient metasurface (center) and two artificial metals (left and right sides). b) Measured Re[*E_z_*] distributions on a plane 50 µm underneath the artificial metal at right‐hand side as the metasurface is shined by a normally incident LCP beam (top panel) or a RCP beam (bottom panel) at 0.4 THz. All fields are normalized against the maximum values in the corresponding patterns. c) Dispersion relations of SSP modes supported by the artificial metal (unit cell shown in the inset), obtained by simulations (solid line) and near‐field measurements (stars). Here, *D* = 83.3 µm, *p* = 166.6 µm, and *L* = 666.4 µm. d) Measured and simulated |*E_z_*|^2^ on the artificial metal at different frequencies excited by our PB meta‐coupler (red symbols and lines) and a conventional grating coupler (black symbols and lines), under the same excitation conditions. e) Schematic of our THz near‐field scanning system for SSP characterizations.

With the PB meta‐atom at hand, we now design a meta‐coupler to achieve high‐efficiency THz SSP excitations. Since metals do not support natural SPs in the THz regime, we first design an “artificial metal” that can support SSPs at THz frequencies, which is a gold film drilled with a periodic array of subwavelength air holes (diameter *D* = 50 µm) with a 60 µm‐thick quartz layer put on its top surface (see inset to **Figure** **3**c). Figure 3c depicts the FEM simulated dispersion relation of the SSP mode supported by such an “artificial metal.” We next use our PB meta‐atom to design a meta‐coupler with phase gradient *ξ* = 2*π*/*L* = 1.14*k*_0_ (lattice constant of super cell *L* = 666.4 µm), that can best match the wave vector of the SSP mode on the “artificial metal” at 0.4 THz (the center frequency of the first high‐efficiency band, see Figure 2d). To achieve this end, we arrange identical PB meta‐atoms in a periodic lattice (with periodicity *P* = 166.6 µm), but with their orientation angles rotated successively with a constant step of Δ*φ* = 45.6° along the *x* direction. According to the PB mechanism,^[^ ^50^, ^51^ ^]^ such a metasurface must exhibit the following phase distribution

(2) $$\begin{array}{c} \Phi^{\sigma }\left(x,y\right)=\Phi_{0}+\sigma \xi \cdot x \end{array}$$

under the excitation of a CP wave with spin‐*σ* (*σ* = +, left circular polarization; *σ* = –, right circular polarization). Here, the phase gradient is determined by *ξ* = 2Δ*φ*/*P* = 9.54 mm.

With the meta‐coupler and “artificial metal” both designed, we then fabricated a device by jointing the meta‐coupler with two pieces of “artificial metals” (see Figure 3a). Shining the meta‐coupler by normally incident CP THz beams with different chirality at 0.4 THz, we employed a THz near‐field mapping technique to characterize the SSP signals excited on the system (see experimental setup in Figure 3e). In our measurement, a probe is placed on a plane 50 µm below the sample moving freely to map the local electric field distribution. The air holes drilled on the “artificial metal” allow the excited SSP wave leaking through the sample, which is then mapped by the THz near‐field probe. Figure 3b depicts the measured Re[*E_z_*] field pattern at 0.4 THz on the reference plane below the right‐hand‐sided artificial metal. When the meta‐coupler is shined by an LCP beam, we find a well‐defined SSP beam generated on the “artificial metal” propagating along +*x* direction (upper panel in Figure 3b). Meanwhile, if the excitation changes to an RCP beam, the excited SSP flows along the opposite direction, as demonstrated by our numerical simulations (see Section A, Supporting Information). From the measured Re[*E_z_*] field pattern, we can quantitatively determine the parallel wave vector of the launched SSP, which is *k*_ssp_ ≈ 1.14*k*_0_ at 0.4 THz, in excellent agreement with the FEM result. Repeating the same measurements at different frequencies, we then experimentally obtain the dispersion relation of the excited SSP modes on the artificial metal, which match well with the FEM‐calculated dispersion (see Figure 3c). FEM simulations are performed on all the cases experimentally studied, which agree well with the measured results.

Having demonstrated the capability of our meta‐coupler to excite SSPs, we now quantitatively evaluate the absolute working efficiency of our scheme, adopting both FEM simulations and near‐field measurements. In our calculations, we numerically integrate the total powers carried by the excited SSP beam and the input CP beam, and then define the ratio between them as the absolute working efficiency of our coupler. The efficiency, thus, calculated reaches 60% at 0.4 THz (see calculation details in Section A, Supporting Information), which is remarkable since it already takes the absorption into account. It is still lower than the bare efficiency of the meta‐atom (≈80%, see Figure 2d), since the designed “artificial metal” only supports SSPs with transverse‐magnetic (TM) polarization while the incident CP wave contains both transverse‐electric (TE) and TM components^[^ ^57^ ^]^ (see more discussions in Section B, Supporting Information). Nevertheless, such a high performance for THz SSP excitation is rarely reported in literature, which can be further improved by reducing the material losses.^[^ ^57^ ^]^ Unfortunately, we cannot use this approach to determine the device efficiency in our experiments. Alternatively, we measured/simulated the E‐field intensities (|*E_z_*|^2^) of the SSPs excited by our meta‐coupler and a conventional grating coupler (see Section C, Supporting Information, for sample picture) of the same size under exactly the same condition, and compared the two results in Figure 3d. Clearly, our meta‐coupler exhibits much higher efficiencies than the grating coupler inside the working band of this device (0.35–0.45 THz). Outside the working band, since our meta‐coupler exhibits a fixed phase gradient not matching well with the wave vectors of SSPs at other frequencies, the SSP excitation efficiency gradually decreases. At the center frequency 0.4 THz, our experiments show that the intensity of SSP (proportional to |*E_z_*|^2^) excited by our meta‐coupler is about 6.25 times higher than that excited by the grating coupler. FEM simulations reveal that the SSP excitation efficiency of the grating coupler is ≈11% at 0.4 THz (see Section C, Supporting Information). Therefore, the efficiency ratio between two devices is about 5.45 at 0.4 THz, evaluated with FEM simulations based on power integrations. This calculated value (5.45) is quite close to that (6.25) obtained in our experiments based local‐field measurements, which collectively demonstrate the performance enhancement of our meta‐device.

### 2.3 High‐Efficiency Meta‐Device Achieving SSP Excitations and Chirality‐Locked Wavefront Reshaping

**Figure 4:**
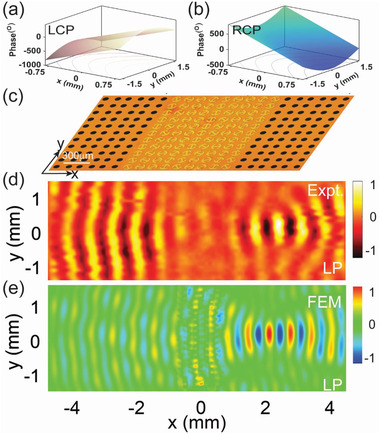
Characterizations on the THz meta‐device enabling SSP excitations and wavefront reshaping simultaneously. a,b) Reflection‐phase profiles encoded in the proposed chirality‐modulated metasurface, as shined by normally incident a) LCP wave and b) RCP wave. c) Picture of part of the fabricated sample consisting of a PB meta‐device connected with two THz artificial metals. d) Measured and e) simulated Re[*E_z_*] distributions on a plane 50 µm underneath the sample, as the central meta‐device is shined by a normally incident LP beam at 0.4 THz. Here, all fields are normalized against the corresponding maximum values in the patterns.

We proceed to demonstrate our second meta‐device that can achieve SSP excitation and wavefront reshaping simultaneously. As depicted in **Figure** **4**c where the sample picture is shown, our meta‐coupler is still constructed by the same PB meta‐atom, but exhibiting a different distribution of orientation angles. Specifically, each row inside the meta‐coupler still consists of a periodic array of PB meta‐atoms exhibiting the same phase slope (*ξ* = 9.54 mm), indicating that each row can convert an impinging CP beam into a SSP with the same wave vector. However, different rows inside the meta‐coupler possess different initial orientation angles (see the first meta‐atom in each row in Figure 3), meaning that the SSP waves excited by different rows can have different phases. The interferences between these SSP waves, excited by different rows, can thus form a new SSP beam with a desired wavefront, dictated by the distribution of the initial phase along the *y* direction.

To illustrate our idea, we follow the above strategy to design a meta‐device assuming that the initial phase exhibits a focusing profile along the *y* direction for LCP excitation. Considering the incidence‐spin dependence of the PB phase, we find that our device should exhibit the following phase distribution

(3) $$\begin{array}{c} \Phi^{\sigma }\left(x,y\right)=\phi_{0}+\sigma \xi \cdot x-\sigma k_{\mathrm{ssp}}\cdot \left(\sqrt{y^{2}+F^{2}}-F\right) \end{array}$$

for spin‐*σ* CP excitation with *F* = 1.5 mm denoting the focal length. Here, *ξ* equals to *k*_ssp_ of the SSP at 0.4 THz. Figure 4a,b illustrates the phase profiles of the designed sample under LCP and RCP excitations, respectively. Obviously, our meta‐coupler is expected to convert a normally incident LCP wave into a SSP beam with a focusing wavefront flowing on the right‐hand‐sided artificial metal, but will convert a RCP beam to a SSP flowing on the left‐hand‐sided artificial metal exhibiting a “defocusing” wavefront. We retrieved the orientation angles of all meta‐atoms according to Equation (3), and then fabricated out the sample together with two guiding‐out artificial metals (see Figure 4c for the sample picture). Shining the meta‐coupler by a LP TH beam under normal incidence, we adopted the same near‐field mapping technique to characterize the field patterns of excited SSPs on the surfaces of two artificial metals. As depicted in Figure 4d,e, our measured near‐field patterns do clearly show that a SSP beam is generated on the artificial metal at the right‐hand side that is then focused to a point, and an SPP beam with a diverging wavefront is generated on the artificial metal at the left‐hand side. We also performed FEM simulations to study the near‐field patterns generated on the artificial metals as the central meta‐coupler is shined by a LP wave at 0.4 THz. The computed patterns depicted in Figure 4e clearly demonstrated the expected functionality of our device, which is in reasonable agreement with the experimental results depicted in Figure 4c. We also numerically evaluated the working efficiency of the meta‐device, and found that it reaches 40% at 0.4 THz (see more details in Section D, Supporting Information).

### 2.4 Bifunctional Meta‐Device Achieving SSP Excitation and Chirality‐Delinked Wavefront Reshaping

While the meta‐device realized in last subsection successfully achieved SSP excitation and wavefront reshaping simultaneously, the two wavefront reshaping functionalities, however, are not independent but rather tightly linked together. The inherent reason is that the PB phases generated under CP excitations with different chirality are strictly opposite with each other. In this subsection, we further realize a meta‐device that can achieve SSP excitations with chirality‐delinked wavefronts. To achieve this goal, we need to find a series of meta‐atoms with reflection‐phases well controlled, independent of the chirality. As we reexamine the reflections of a meta‐atom exhibiting the Jones’ matrix *R* in responses to CP excitations, we find that the anomalous reflection coefficient is explicitly given by

(4) $$\begin{array}{c} {\tilde{r}}_{a}=\left(\frac{r_{uu}-r_{vv}}{2}\right)e^{i\sigma 2\varphi }=R_{a}e^{i\left(\phi_{\mathrm{res}}+\phi_{\mathrm{PB}}\right)} \end{array}$$

where, in addition to the *σ*‐dependent PB phase *ϕ*_PB_ = 2*σ* · *θ*, a new phase *ϕ*_res_ = arg(*r_uu_* – *r_vv_*) appears, which is obviously dictated by the structure rather than the orientation angle *θ* and the incident chirality *σ*. We find that *ϕ*_res_ can be strongly modulated simply via tuning the opening angle *α* of the meta‐atom (see Figure 2). Meanwhile, changing *α* does not significantly decrease the working efficiency *R*_a_ of the meta‐atom at 0.4 THz, since the two magnetic resonances for two linear polarizations are shifted in a parallel way so that the whole structure still behaves as a half‐wave plate approximately (see more details in Section E, Supporting Information).

Such resonance‐induced phase *ϕ*_res_ offers us the opportunities to realize any desired chirality‐delinked phase distributions $\Phi^{\sigma }\left(x,y\right)=\phi_{\mathrm{res}}\left(x,y\right)+\phi_{\mathrm{PB}}^{\sigma }\left(x,y\right)$, simply through tuning the orientation angle (*θ*) and the geometric parameter (*α* ) for each individual meta‐atom inside the meta‐device. In contrast, since only PB phases are adopted in the scheme proposed by Xiao et al.,^[^ ^55^ ^]^ two SP‐control effects enabled by the metasurfaces designed with that scheme are still inherently correlated with each other. The bifunctionalities on controlling SPs are only meaningful inside a particular region on the metal surface, thanks to the chirality‐induced real‐virtual image exchange.

**Figure 5:**
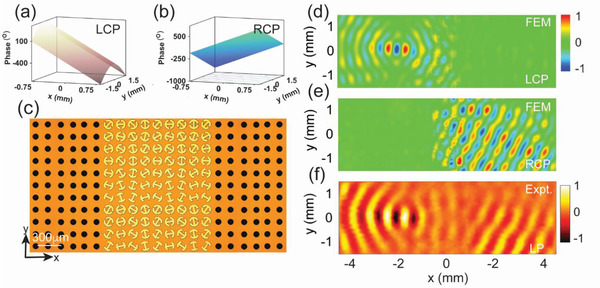
Characterizations on the THz meta‐device enabling SSP excitations and chirality‐delinked wavefront re‐shaping simultaneously. a,b) Reflection‐phase profiles encoded in the proposed chirality‐modulated metasurface, as shined by normally incident a) LCP wave and b) RCP wave. c) Picture of part of the fabricated sample consisting of a PB meta‐device connected with two artificial metals. Simulated Re[*E_z_*] patterns on a plane 50 µm underneath the sample, as the meta‐device is shined by a normally incident d) LCP beam or e) RCP beam at 0.4 THz, respectively. f) Measured Re[*E_z_*] pattern on a plane 50 µm underneath the sample, as the meta‐device is shined by a normally incident LP beam at 0.4 THz. Here, all fields are normalized against the corresponding maximum values in the patterns.

We now take a specific example to illustrate our idea. Assume that our meta‐device exhibits the following phase distributions (see **Figure** **5**a,b) under excitations of CP waves with two chirality

(5) $$\begin{array}{c} \left\{\begin{array}{c} \Phi^{+}\left(x,y\right)=\phi_{0}-\xi \cdot x-\xi \cdot \left(\sqrt{y^{2}+F^{2}}-F\right)\\ \Phi^{-}\left(x,y\right)=\phi_{0}+\xi \cdot x-\xi \cdot y\sin\theta_{r} \end{array}\right) \end{array}$$

where *ξ* equals to *k*_ssp_ of the SSP at 0.4 THz. Obviously, the meta‐device is expected to convert a LCP beam into a SSP beam focused to a focal point at the right‐hand side, and convert an RCP beam into a SSP that is then deflected off the *x* direction on the left‐hand side. Retrieving the *θ*(*x*,*y*) and *α*(*x*,*y*) distributions based on Equation (5) (see more details in Section F, Supporting Information), we then fabricated out the meta‐device (see sample image in Figure 5c) and performed the THz near‐field experiments. Figure 5f depicts the measured near‐field patterns on a reference plane 50 µm below the sample, as the meta‐coupler was shined by a normally incident LP beam at 0.4 THz. The measured patterns clearly verified our predictions. We next performed full wave simulations to study the near‐field patterns generated on the meta‐device, as it is shined by normally incident THz beam with different spins. Figure 5d,e shows that the numerical results are in the reasonable agreement with experiments and theoretical predictions. The absolute working efficiencies of these two functionalities are also numerically evaluated, which are 30% and 35% for focusing and deflection, respectively (see more details in Section G, Supporting Information). These efficiencies are lower than that of the pure SPP excitation efficiency (60%), which is quite understandable since combining two functionalities together can increase the scatterings of driven surfaces waves as they flow on the meta‐device and are guided out to the artificial metals.^[^ ^58^, ^59^ ^]^ In addition, missing the TE‐polarized SSPs on the guiding‐out artificial metal is also an issue degrading the performance.^[^ ^57^ ^]^

## 3 Conclusion and Outlook

In conclusion, we propose a new strategy to design high‐efficiency meta‐devices to achieve SSP excitations and wavefront tailoring simultaneously, and verify our idea based on three successive experiments in the THz regime. After experimentally demonstrating the SSP excitation functionality of a THz meta‐coupler constructed by a carefully designed high‐efficiency PB meta‐atom, we employed the same PB meta‐atom to design/fabricate a meta‐device exhibiting focusing and gradient phase distributions along two cross directions, and that experimentally it can excite SSPs with focusing or defocusing wavefronts, as shined by CP waves with different chirality. Finally, combining resonance and PB mechanisms, we experimentally demonstrate the third meta‐device that can achieve SSP excitations with wavefronts independently engineered, as shined by CP waves with different chirality. All experiments are in agreement with full wave simulations. Our results establish a new platform to control THz near‐fields with ultracompact devices, which may find many applications in integration‐optics research. For example, to detect certain signals arising from interactions between lights and emitters (e.g., molecules or quantum dots) placed on a metal surface, one can use our scheme to couple light into SSPs that are subsequently focused to that spot. Compared to the direct‐excitation scheme, signals obtained via our scheme are background‐free with magnitudes significantly enhanced. In addition, one can also use our scheme to couple propagating waves directly into on‐chip optical waveguides with high efficiencies (see more details in Section H, Supporting Information). Finally, meta‐devices constructed with our scheme, exhibiting both small feature sizes and multiple functionalities, are highly desired in future integration‐optics applications.

## Conflict of Interest

The authors declare no conflict of interest.

## Supporting information

Supporting Information
