# Supplementary material for: Excite Spoof Surface Plasmons with Tailored Wavefronts Using High‐Efficiency Terahertz Metasurfaces
Source: Adv Sci (Weinh). 2020 Aug 5;7(19):2000982. doi: 10.1002/advs.202000982 (PMC7539192; doi:10.1002/advs.202000982)
Supplement: Supplementary file 1 — Supporting Information [file ADVS-7-2000982-s001.pdf]

2020 Wiley-VCH GmbH

ADVANCED SCIENCE
Open Access

Supporting Information

for *Adv. Sci.,* DOI: 10.1002/advs.202000982

Excite Spoof Surface Plasmons with Tailored Wavefronts Using High-Efficiency Terahertz Metasurfaces

Zhuo Wang, Shiqing Li, Xueqian Zhang, Xi Feng, Qingwei Wang, Jiaguang Han, Qiong He, Weili Zhang, Shulin Sun, and Lei Zhou**

Supporting Information

Excite spoof surface plasmons with tailored wavefronts using high-efficiency terahertz metasurfaces

Zhuo Wang[1†], *Shiqing Li*[2†], *Xueqian Zhang*[3†], *Xi Feng*[3], *Qingwei Wang*[3], *Jiaguang Han*[3], *Qiong He*[1,4,5], *Weili Zhang*[6], *Shulin Sun*[2,4*], *Lei Zhou*[1,4,5*]

[1]State Key Laboratory of Surface Physics and Key Laboratory of Micro and Nano Photonic Structures (Ministry of Education), Fudan University, Shanghai 200433, China.
[2]Shanghai Engineering Research Center of Ultra-Precision Optical Manufacturing, Green Photonics and Department of Optical Science and Engineering, Fudan University, Shanghai 200433, China.
[3]Center for Terahertz waves and College of Precision Instrument and Optoelectronics Engineering, Tianjin University and the Key Laboratory of Optoelectronics Information and Technology (Ministry of Education), Tianjin 300072, China.
[4]Academy for Engineering and Technology, Fudan University, Shanghai 200433, China.
[5]Collaborative Innovation Center of Advanced Microstructures, Nanjing 210093, China.
[6]School of Electrical and Computer Engineering, Oklahoma State University, Stillwater, Oklahoma 74078, USA.

E-mail: sls@fudan.edu.cn, phzhou@fudan.edu.cn
[†] These authors contributed equally to this work.

Keywords: (metasurface, spoof surface plasmon, wavefront, plasmonic coupler, near field)

A. Numerical studies on the meta-device examined in Figure 3 of the main-text

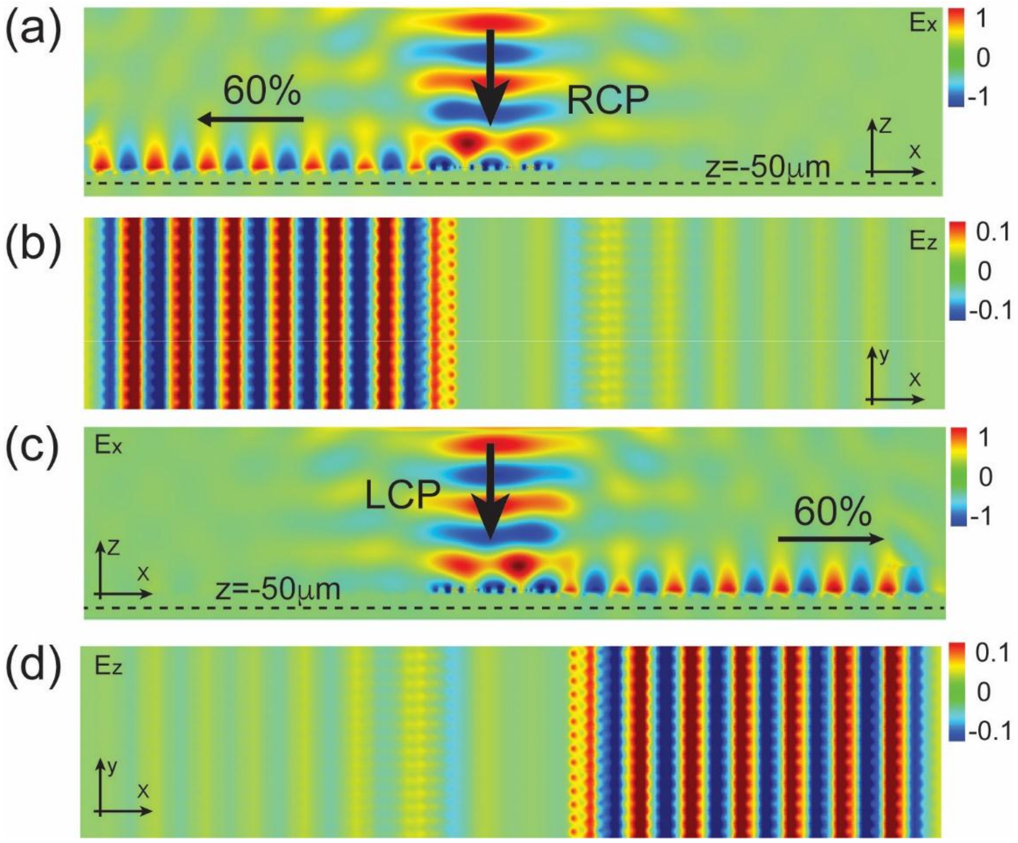

Figure S1. FEM simulated Re[E_x] field patterns on (a, c) the *x*-*z* plane (with *y*=0) and Re[E_z] field patterns on (b, d) *x*-*y* plane (50μm below the artificial metal) of the meta-device depicted in Figure 3 of the main-text, as the center region is shined respectively by a (a, b) RCP and (c, d) LCP Gaussian beam at 0.4 THz. In our simulations, we use a source current on the upper boundary to illuminate the CP wave on the meta-device and perfectly matched layers (PMLs) along ±x direction to dissipate the guided-out SSPs on the artificial metal. Periodic boundary conditions are applied along y direciton. We numerically integrated the powers carried by the guided-out SSP beam and the input CP beam, respectively. The ratio between them is defined as the absolute working efficiency of our meta-coupler, which is 60% at 0.4 THz. The length of the simulated PB meta-coupler is 1499.4 μm, the same as that of the sample studied in Figure 3.

B. Polarization conversion effect in the SSP excitation process

Since the input CP wave contains TE and TM polarized components with equal amplitudes, it is intriguing to note that the SSP excitation efficiency achieved with our meta-coupler (see Figure 3 in the main-text) can exceed 50% considering that the excited SSPs are only of the TM polarization. Such an intriguing result is caused by a polarization conversion effect in this process, as detailed in the following.

As schematically depicted in Figure S2, the conversion from incident CP propagating wave (PW) to SSP actually contains two steps: 1) a conversion from CP PW to TE and TM polarized driven surface wave (SW) bounded at the metasurface coupler; 2) a conversion from the driven SW to eigen SSP flowing on the designed artificial metals placed at two sides of the meta-coupler. In the first process, the polarization of impinging wave is well perserved, implying that the generated driven SW still possess nearly equal components of TE and TM polarizations (as schematically depicted in Figure S2a). However, in the second process (i.e., such driven SWs pass across the coupler boundary to flow as eigen SSPs on the artificial metals), we find that while the TM polarized driven SW can efficiently couple into the TM eigen-SSP, the TE-polarized driven SW, however, can convert a large portion of its energy into TM-polarized eigen-SSP on the artificial metals (as schematically depicted in Figure S2b). It is such a polarization conversion effect that finally pushes the PW-SSP conversion efficiency to a value higher than 50% (60% in present THz devices and even higher value in microwave devices as demonstrated in Sci. Rep. 7, 1354 (2017)).

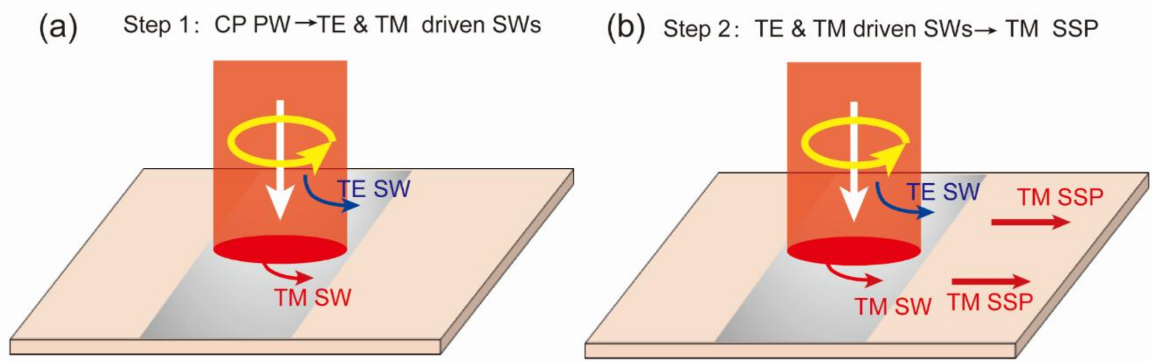

Figure S2. Two fundamental steps in the conversion process from free-space CP PW to near-field SSP.

As the two processes are coupled together, it is difficult to directly see such a polarization conversion effect. To clearly reveal the physics, we purposely designed a THz artificial metal that supports both TE and TM SSPs at the working wavelength (see Figure S4), and put two such artificial metals on both sides of the meta-coupler (adopted in Figure 3 of the main-text)

to form a model system for numerical simulation (see Figure S3). We employed FEM simulations to study the transmissions through the junction of SSPs with different polarizations launched on the left-hand-sided artificial metal. As the input TE polarized SSP passes through the meta-coupler region, we find that strong TM SSP signal appears on the right-hand-sided artificial metal (see Figure S3b where the E_z component is depicted) but the TE-SSP signal nearly disappears (see Figure S3c where the E_y component is depicted). In contrast, as we change the polarization of input SSP to TM polarization, we obtain strong TM SSP signals and very weak TE SSP signals on the right-hand-sided artificial metal (see Figure S3e-S3f)). These results unambiguously demonstrate the polarization conversion effect mentioned above, well explaining why the finally achieved PW-SSP conversion efficiency can exceed 50%.

Finaly, we briefly discuss the inherent physics. Here, TE SSP cannot propagate inside the region occupied by the PB meta-coupler, but TM SSP can propagate inside it (see Figure S3), caused by different boundary conditions for waves with two different polarizations. Intriguingly, since our PB meta-atom is anisotropic, once it is rotated, it can convert some of the TE-polarized wave to TM-polarized one after transmission. Therefore, after passing through the region occupied by the meta-coupler, a considerable portion of TE-SSP has been converted to TM-SSP, as illustrated in Figure S3.

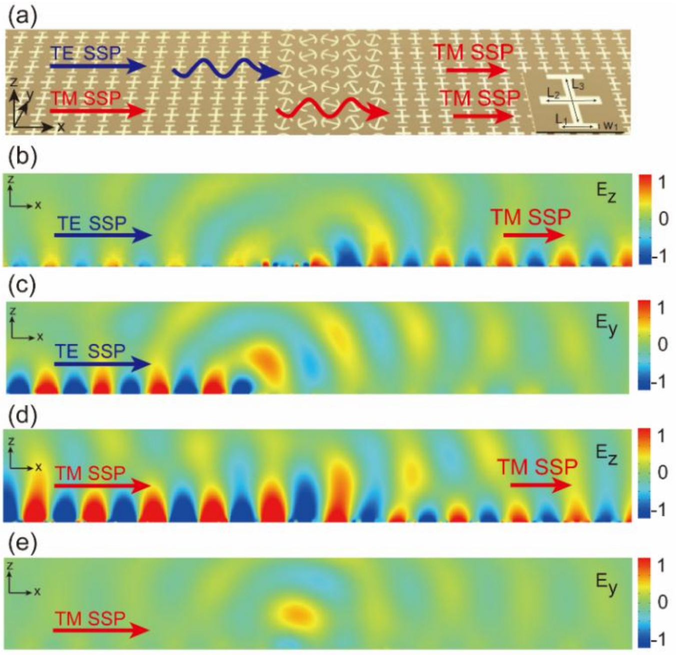

Figure S3. (a) Schematic of the PB meta-coupler with a new artificial metal supporting both TE and TM SSPs. Simulated (b, d) Re[E_z] and (c, e) Re[E_y] field pattern inside the PB meta-

coupler as the (b, c) TE and (d, e) TM SSPs is respectively excited at the left side of the device.

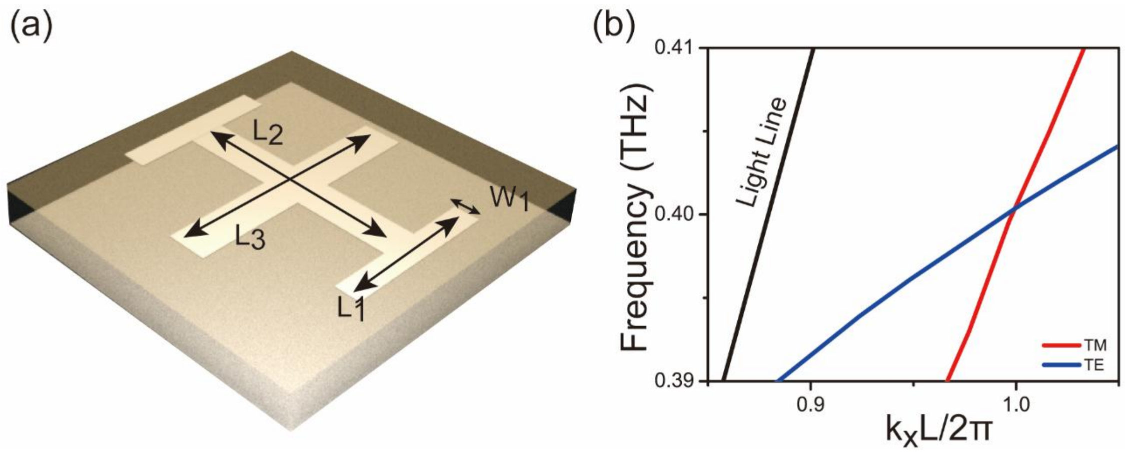

Figure S4. (a) Schematic of the unit cell of an artificial metal supporting both TE and TM polarized SSPs, which is composed of a metallic microstructure and a gold film separated by a 60 μm-thick quartz layer. Here, $L_1 = 53\ \mu m$, $L_2 = 86.6\ \mu m$, $L_3 = 92\ \mu m$ and $W_1 = 23.2\ \mu m$. (b) Dispersion relations of the eigen TE and TM SSPs supported by such artificial metal.

C. Sample image of the fabricated grating coupler and the numerically computed SSP excitation efficiencies of two different couplers

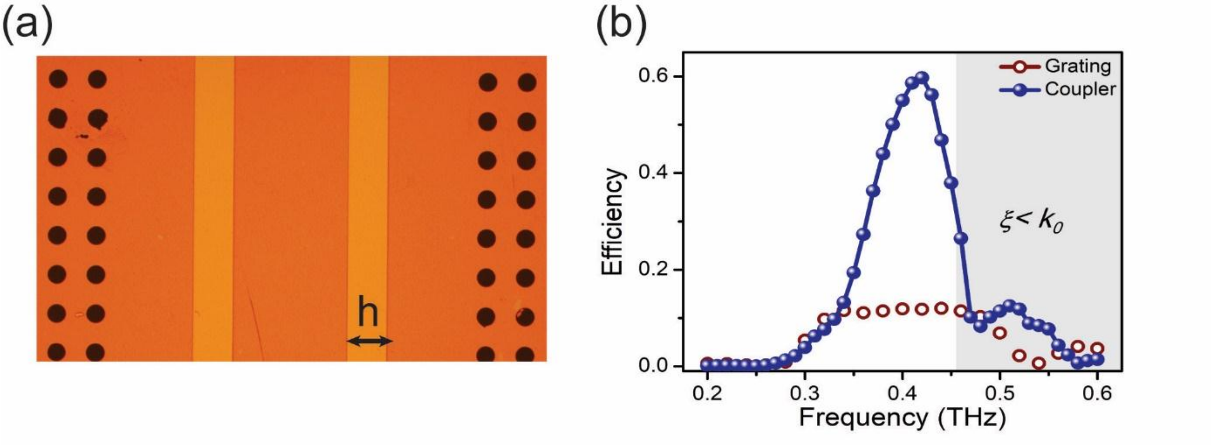

Figure S5. (a) Top-view picture of part of the fabricted grating coupler, composed by two 167μm-wide gold stripes of the periodicity 658. 76μm and a contineous gold mirror, separated by a 60μm-thick quartz spacer ($\varepsilon_r = 3.9 + 0.11i$). The total width of the grating coupler is 1449.4μm, identical to that of the PB meta-coupler. (b) The simulated SSP excitation efficiencies of the grating coupler as shown in (a) and the PB meta-coupler as shown in Figure 3 of the main-text. Note that the SSPs can still be excited even at frequencies with $\xi < k_0$ (gray region in (b)), simply because both couplers exhibit finite sizes. Here, the simulation conditions (i,e., the beam size, the boudnary conditions, the artificial metals) are totally same as those in Figure S1. We numerically integrated the powers carried by the guided-out SSP beam and the input CP beam, respectively. The ratio between them is defined as the absolute working efficiency of the grating coupler

D. Simulation details of the SSP focusing effect in Figure 4 of the main-text

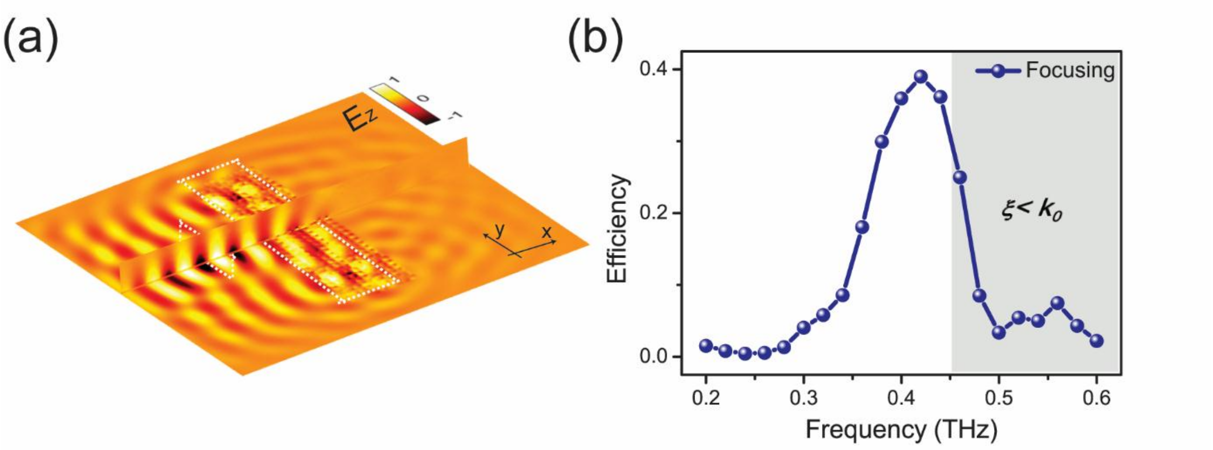

Figure S6. (a) Simulated Re[E_z] field patterns on the *x*-*y* plane (with z=0) and on the *x*-*z* plane (with *y*=0) in the meta-coupler as studied in Figure 4, while the meta-coupler is shined by a LCP beam at 0.4 THz. (b) Simulated SSP focusing efficiency of the PB meta-coupler as a function of frequency. Here, we integrate the power of the focused SSP within a rectagular region at the its focal plane (see (a)), and integrate the power illuminated on the meta-coupler of the size $1500\times 5160 \mu m^2$. The ratio between these two values is defined as the working efficiency. Note that our device still works even at frequencies with $\xi < k_0$ (gray region in (b)), simply because the meta-coupler exhibits a finite size. Here, a scouce current boundary is used at the upper boundary (along +z direction) to illuminate CP on the meta-coupler and PMLs are applied at the boundaries along ±x and ±y directions to absorbe the out-going SSPs on the artificial metal.

E. Broadband half-wave plate effect of the proposed PB meta-atoms with different opening angles

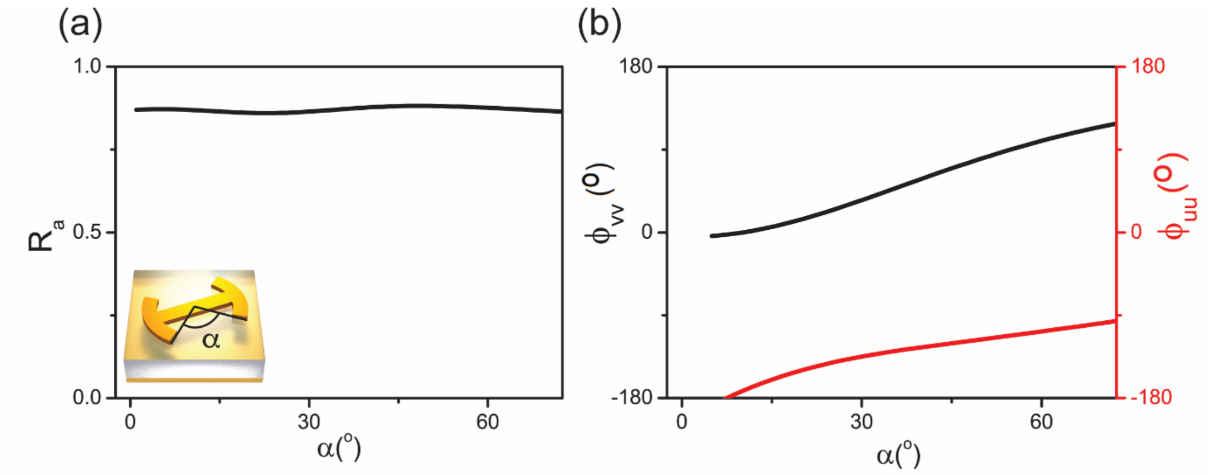

Figure S7. (a) The power efficiency of the anomalous mode $R_a = |r_{uu} - r_{vv}|^2 / 4$ and (b) the reflection phases (φ_{uu} and φ_{vv}) for a series of PB meta-atoms depicted in Figure 2 with different α, obtained by numerical simulations at 0.4 THz, exhibiting excellent half-wave plate properties.

F. Retrieving the $\theta(x,y)$ and $\alpha(x,y)$ parameters of the bi-functional meta-device in Figure 5 of the main-text

The desired phase profiles of the PB meta-device in Figure 5 reads:

$$\begin{cases}\Phi^{+}(x,y)=\phi_0-\xi\cdot x-\xi\cdot(\sqrt{y^2+F^2}-F)\\ \Phi^{-}(x,y)=\phi_0+\xi\cdot x-\xi\cdot y\sin\theta_r\end{cases}. \qquad \text{(S1)}$$

To build such a device, we need to sort out all meta-atoms with appropriate θ and α. For our meta-atoms, their total spin-dependent phases contain two parts, the resonance phase $\phi_{\rm res}$ and the PB phase $\sigma\phi_{\rm PB}$. Therefore, we have

$$\Phi^{\sigma}(x,y)=\phi_{\rm res}(x,y)+\sigma\phi_{\rm PB}(x,y), \qquad \text{(S2)}$$

where $\phi_{\rm res}(x,y)$ and $\phi_{\rm PB}(x,y)$ describe the desired values of resonance phases and PB phases for meta-atoms located at different positions. Substituting Eq. (S1) into Eq. (S2), we can obtain the desired $\phi_{\rm res}(x,y)$ and $\phi_{\rm PB}(x,y)$ distributions, based on which we can select the meta-atom (with appropriate rotation angle θ and opening angle α) located at position (*x*, *y*).

To determine the two distributions: $\theta(x,y)$ and $\alpha(x,y)$, we need to know how $\phi_{\rm PB}$ and $\phi_{\rm res}$ vary against θ and α. According to the PB mechanism, we get

$$\phi_{\rm PB}=2\theta, \qquad \text{(S3)}$$

rigorously. Meanwhile, we can use the definition

$$\phi_{\rm res}=\arg(r_{uu}-r_{vv}), \qquad \text{(S4)}$$

to compute how the resonance-phase $\phi_{\rm res}$ varies against α, simply through putting r_{uu} and r_{vv} calculated for meta-atoms with different α into Eq. (S4). We call such a phase as the resonance phase based on far-field (FF) calculations. The computed $\phi_{\rm res}^{\rm FF}\sim\alpha$ relation is depicted in Figure S8(b) as a black line.

However, we note that the meta-device presented in Figure 5 of the main-text works for SW manipulations. Each row of such a device is a gradient meta-coupler to convert normally incident propagating wave into a SSP, but with different initial phase. It is such a phase, defined by the near-field (NF) phase ϕ_{res}^{NF} , working to modulate the wave-front the whole SSP beam. Therefore, it is more accurate to use the $\phi_{res}^{NF} \sim \alpha$ relation to select our meta-atoms with different geometric structures (i.e., α). The calculation method is schematically shown in Figure S8a. Normally shine a circular-polarized (CP) THz wave onto PB meta-couplers exhibiting the same phase gradient $\xi = k_{ssp}$ composed by the meta-atoms with different α , we can generate SSP beams on the artificial metal. The phases of these SSP beams are then defined as ϕ_{res}^{NF} . The calculated $\phi_{res}^{NF} \sim \alpha$ relation is shown as a red line in Figure S8b. We choose the references of two phases by requiring $\phi_{res}^{NF} = \phi_{res}^{FF}$ for $\alpha = 38^{\circ}$. We note that ϕ_{res}^{NF} exhibits slight differences with ϕ_{res}^{FF} , which can be attributed to multiple scatterings of SSPs inside the meta-coupler.

Based on Eq. (S3) and the $\phi_{res}^{NF} \sim \alpha$ curve as shown in Figure S8b, we finally obtain the $\theta(x, y)$ and $\alpha(x, y)$ distributions of the desired device, which are shown in Figure S8c and 8d, respectively. With these two distributions, we then design our meta-device and fabricate it out.

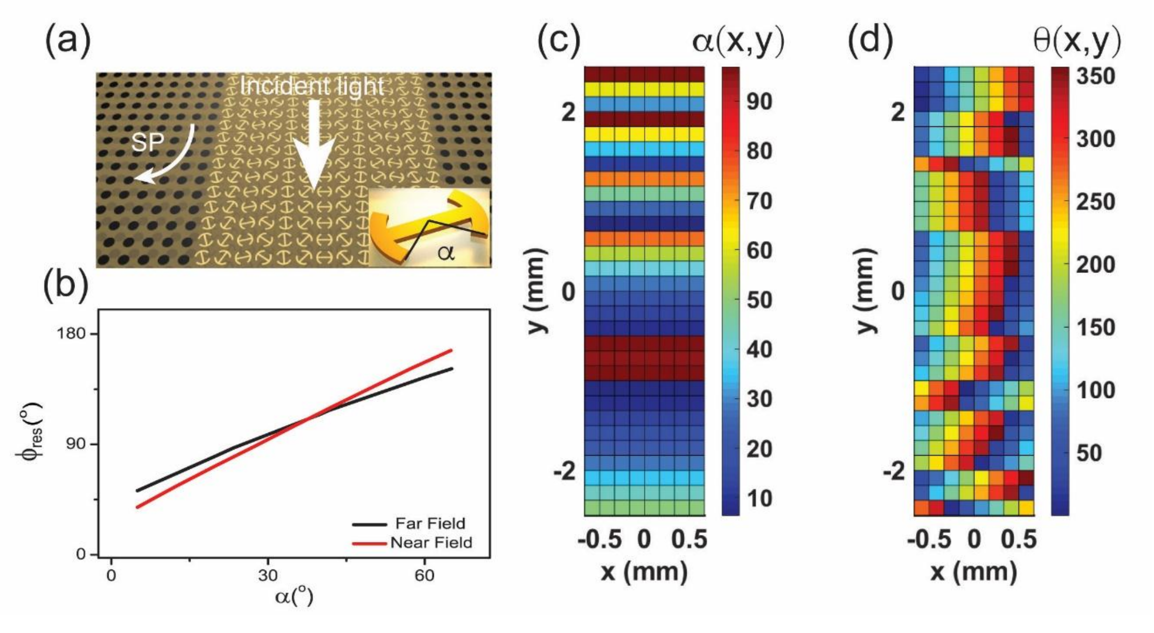

Figure S8. (a) Schematic on retrieving the NF phases ϕ_{res}^{NF} of our meta-atoms. (b) Resonance phases versus the opening angle α of the meta-atom, obtained by NF and FF calculations. (c,

d) Distributions of $\alpha(x, y)$ and $\theta(x, y)$ of the fabricated meta-device studied in Figure 5 of the main-text.

G. Working efficiency of the bi-functional meta-coupler in Figure 5 of the main-text

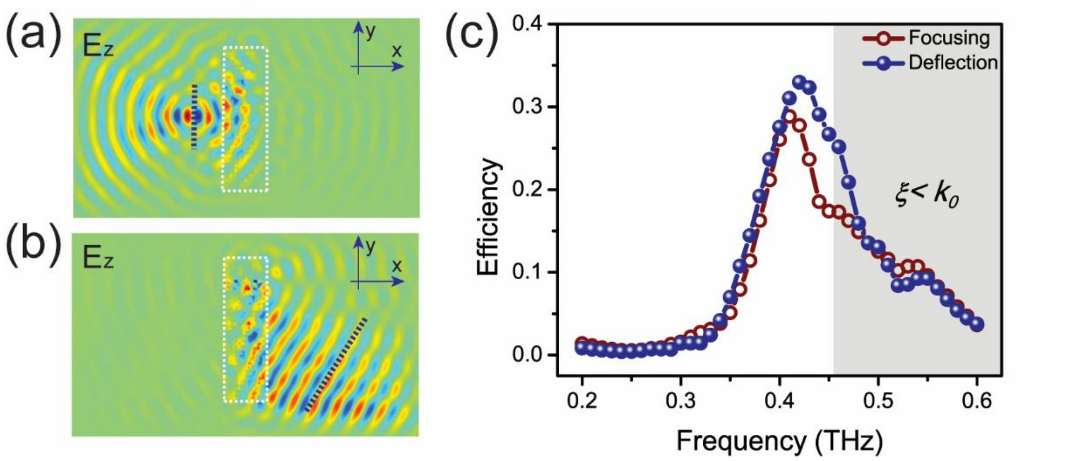

Figure S9. (a, b) Simulated Re[E_z] field patterns on the top surface of the sample studied in Figure 5, as the central meta-coupler is shined respectively by a (a) LCP and (b) RCP beam at 0.4THz. (c) The calculated efficiency spectra of the SSP focusing and deflection effects of our meta-device. In computing the efficiency in (a) (or (b)), we integrate the power carried by the focused SSP (or deflected SSP) passing through the rectangular area (large enough to cover the whole SSP signals) located at the focal plane (or the wavefront plane), and the power carried by the incident beam, and define the ratio between the two values as the working efficiency. Here, the simulation conditions are similar to those in Figure S6.

H. Numerical demonstrations of the practical applications for our meta-coupler.

The proposed meta-coupler for near-field SSP excitation and wavefront engineering can find many photonic applications in THz and other frequency domains, e.g., enhancing light-matter interactions, coupling on-chip optical devices, bio- or chemical sensing, imaging, etc. For example, we have numerically demonstrated that such meta-device can efficiently couple the optical waveguide with its end put at the SSP focal point of the meta-coupler proposed in Figure 4. As shown in Figure S10c and S10d, most of the input CP wave can be efficiently converted to SSP at the focal point and then further coupled into the waveguide. For comparison, the conventional coupling method (e.g., the direct illumination by the input Gaussian beam) is obviously less efficient, which is quite reasonable considering that the cross-section of the waveguide is much smaller than input beam size, as depicted in Figure S10e and S10f.

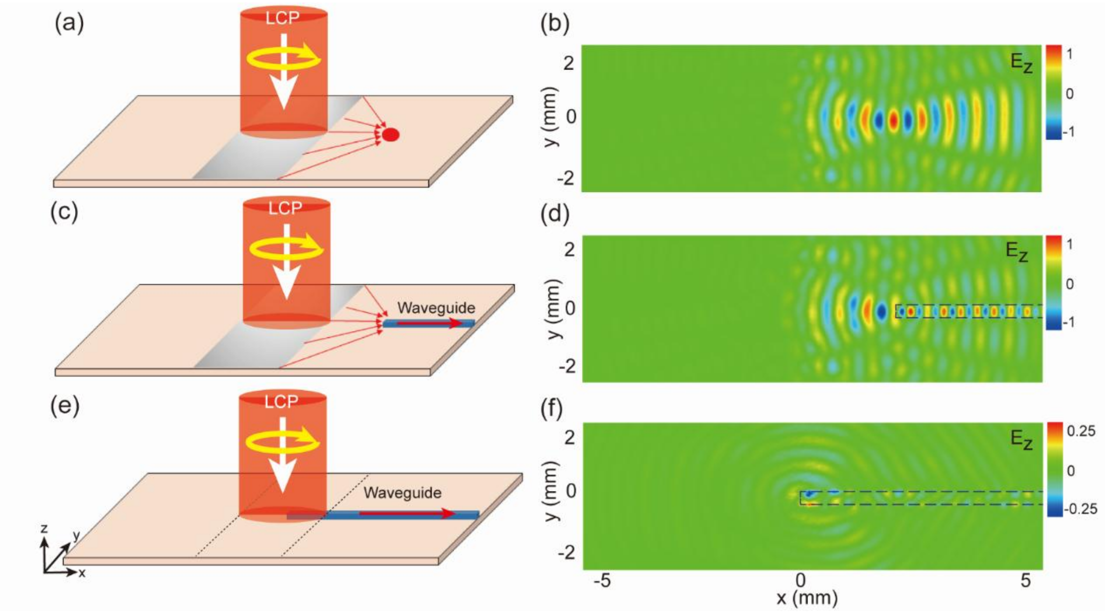

Figure S10. (a, b) SSP focusing effect and (c, d) its coupling with the on-chip optical waveguide by our PB meta-coupler. (e, f) Direct coupling of the on-chip waveguide by the input Gaussian beam.
